# Severity of Depression Predicts Remission Rates Using Transcranial Magnetic Stimulation

**DOI:** 10.3389/fpsyt.2015.00114

**Published:** 2015-09-01

**Authors:** Geoffrey G. Grammer, Andrew R. Kuhle, Caroline C. Clark, Michael N. Dretsch, Kathy A. Williams, Jeffrey T. Cole

**Affiliations:** ^1^Department of Research, National Intrepid Center of Excellence, Walter Reed National Military Medical Center, Bethesda, MD, USA; ^2^Department of Psychiatry, Walter Reed National Military Medical Center, Bethesda, MD, USA

**Keywords:** rTMS, depression, frequency, remission, response

## Abstract

**Background:**

Multiple factors likely impact response and remission rates in the treatment of depression with repetitive transcranial magnetic stimulation (rTMS). Notably, the role of symptom severity in outcomes with rTMS is poorly understood.

**Objective/hypothesis:**

This study investigated the predictors of achieving remission in patients suffering from depression who receive ≥3 rTMS treatments per week.

**Methods:**

Available data on 41 patients treated at Walter Reed National Military Medical Center from 2009 to 2014 were included for analysis. Patients received a range of pulse sequences from 3,000 to 5,000 with left-sided or bilateral coil placement. Primary outcome measures were total score on the Patient Health Questionnaire-9 or the Quick Inventory of Depressive Symptomatology-Self Rated. Remission was defined as a total score less than five, and response was defined as a 50% decrease in the total score on both outcome metrics. Outcomes in patients diagnosed as suffering from mild or moderate depression were compared to those suffering from severe depression.

**Results:**

Of the 41 patients receiving treatment, 16 reached remission and 18 reached response by the end of treatment. Remission rate was associated with the initial severity of depression, with patients with mild or moderate depression reaching remission at a significantly higher rate than those with severe depression. Total number of rTMS sessions or length of treatment was not predictors of remission.

**Conclusion:**

Patients with a baseline level of depression characterized as mild or moderate had significantly better outcomes following rTMS compared to patients with severe depression.

## Introduction

In October 2008, the U.S. Food and Drug Administration (FDA) cleared the use of repetitive transcranial magnetic stimulation (rTMS) for the acute treatment of major depression. Studies cited in support of this decision used the Neuronetics Neurostar TMS Therapy^®^ system with an iron core figure-8 coil at 10 pulses/s, 120% of the motor threshold (MT), 3,000 pulses per treatment, and five treatments per week for 4–6 weeks (1–3). Since FDA approval, several subsequent studies have supported the findings of efficacy of rTMS in treating depression. In a non-industry supported study, George et al. (4) reported similar findings that further substantiate the use of rTMS for treatment of depression (4). A meta-analysis of 18 good or fair quality studies investigating the use of rTMS for treatment-resistant depression reported that rTMS is a reasonable, effective consideration (5). The preponderance of evidence would therefore appear to suggest a beneficial effect of rTMS as a treatment for depression.

To most effectively apply rTMS treatment for depression, extensive research efforts have investigated possible predictors of outcome. Age of the patient and medication refractoriness are two key indicators for response to rTMS treatment, with younger and non-refractory patients responding better to treatment (3, 6). In fact, treatment resistance is a consistently reported predictor of antidepressant response (7–9). Fregni et al. (6) speculated that individuals refractory to pharmacological or other therapies may have a more severe form of depression, which would explain why those individuals have a reduced response to therapy (6). In a limited-scale study, Su et al. (10) reported that patients with less severe depression have a greater response to rTMS treatment (10). In contrast to those results, Lisanby et al. (3) determined that baseline symptom severity had no impact on treatment outcome (3). Numerous other factors have also been suggested as predictors for outcome, including depressive subtype (11), anxiety comorbidity (12, 13), duration of the current depressive episode, and polymorphisms affecting serotonin and glutamate transmission (14, 15).

To answer questions regarding outcome predictors of remission following rTMS treatment, we conducted a retrospective study of patients treated for depression at Walter Reed National Military Medical Center (WRNMMC). Parameters examined included baseline depression levels, total number of sessions, length of treatments, and pulse frequencies.

## Materials and Methods

We included all patients (*N* = 70) previously treated for depression with rTMS at WRNMMC between March 2009 and February 2014 whose data were retained within the patient data management system (PDMS). The population consisted of both females (57.5%) and males (42.5%) with a mean age of 41.0 (SD = 15.5) (Table 1). Variations of treatment delivery occurred due to provider and patient preference. Compliance, and therefore inclusion in this study, was determined to be ≥3 treatments per week.

**Table 1:** **Overall demographic information on all patients included in the retrospective study investigating the use of rTMS as a treatment for depression following mTBI (*n* **=** 41)**.

Age, *M*(SD)	41.0 (±15.5)
Gender^a^	% of sample
Male	17/40 (42.5%)
Female	23/40 (57.5%)
Number of sessions, *M*(SD)	26.0 (±7.4)
Frequency	
3,000 L	66.7%
5,000 L	2.4%
3,500 L and 1,500 R	9.5%
Mixed	21.4%

*^a^The gender data from one individual was missing. Therefore, percentages are based off of an  = 40*.

**Figure 1:**
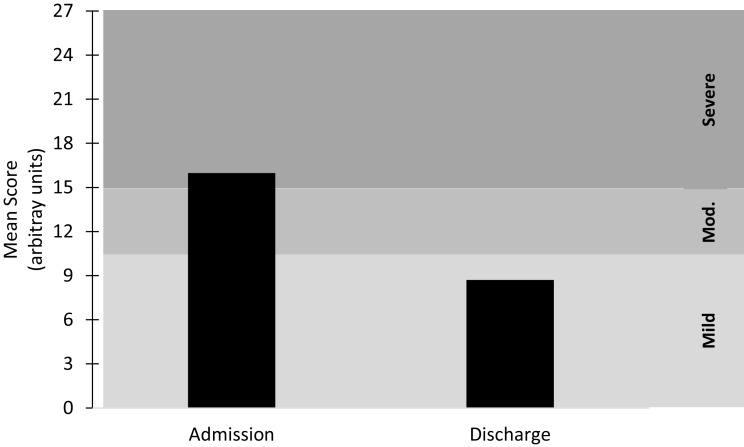
**Mean score at admission and discharge for the combined depression metrics**. Mild ≤ 10; Moderate (Mod.) = 11–15; Severe ≥ 16. *n* = 41.

Forty-six of the 70 participants maintained the compliance standard of three or more treatments per week (66% compliance), while 5 participants were missing outcome data and were removed from the analysis. Therefore, data from 41 patients were included in this study – 20 individuals with mild-to-moderate depression and 21 individuals with severe depression (Figure 1). The current study was performed following review and approval by the WRNMMC Institutional Review Board. The study was conducted in accordance with all Federal laws, regulations, and standards of practice as well as those of the Department of Defense and the Departments of Army/Navy/Air Force.

Prior to beginning rTMS administration, providers administered the Patient Health Questionnaire-9 (PHQ-9) (16) and/or the Quick Inventory of Depressive Symptomatology-Self Rated (QIDS-SR) (17). All patients were treated with NeuroStar TMS Therapy^®^ by Neuronetics. For this study, administration consisted of at least 10 treatment sessions. A 50% decrease in the patients PHQ-9 or QIDS-SR score from baseline defined “response.” A score of <5 for the QIDS-SR and <5 for the PHQ-9 defined remission.

The NeuroStar^®^ device includes a PDMS, which is a self-contained HIPAA-compliant electronic health record. The PDMS is able to store demographic data, dates and parameters of treatment, and scores from rating scales administered during treatment. The following patients were excluded from the study: received <10 treatments, no PHQ-9 or QIDS-SR scores, did not meet the minimal depression criteria for the PHQ-9 (five or greater) or QIDS-SR (six or greater), or did not receive left-sided or bilateral coil placement. Left-sided treatments were administered at 10 Hz, 120% of the MT, 3,000–5,000 pulses per treatment, in 4-s stimulus trains with 26 s rest intervals. Bilateral treatments were administered initially left-sided at 10 Hz, 120% of the MT, 3,500 pulses, in 4-s stimulus trains with 26 s rest intervals followed by right-sided application of 1 Hz, 120% of the MT, 1,500 pulses, in 26-s stimulus trains with 4-s rest intervals. All stimuli were applied 5.5 cm anterior of the MT location corresponding to the abductor pollicis brevis. Data were extracted from the PDMS, de-identified, and entered into an Excel sheet. We extracted all rating scales scores, the frequency of treatment, the number of pulses per treatment, the dates of treatment, and the coil placements during treatment.

Binary logistic regression (backward Wald) was utilized to determine whether the number of session, length of treatments (weeks), pulse frequency or baseline depression scores predicted remission (final outcome score <5) using rTMS. A Chi-squared (χ^2^) test was run to determine differences in depression severity scores (mild-to-moderate versus severe) for remission. Severity was classified by either PHQ-9 or QIDS-SR scale cutoffs as described in Figure 1.

## Results

**Figure 2:**
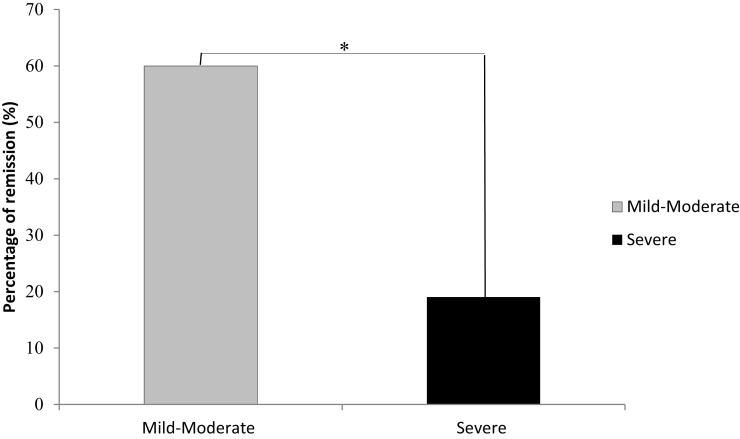
**Remission rates were significantly higher in participants with mild-to-moderate depression than in those individuals initially diagnosed as experiencing severe depression**. *Denotes a significant difference of *p* = .007; mild-moderate, *n* = 20; severe, *n* = 21.

Across all severity levels of depression, 16 of the 41 (38%) patients reached remission by the end of their treatment. In the mild-to-moderate group, 12 of the 20 (60%) achieved remission; in the severe group, 4 of the 21 (19%) achieved remission. Across all severity levels of depression, response rate was reached by 18 of the 41 (43.9%) patients. In the mild-to-moderate group, 11 of the 20 (55%) achieved response; in the severe group, 7 of the 21 (33%) achieved response. Baseline depression severity scores were the only significant predictor of improvement following rTMS treatment (*p* = .008). Patients with mild-to-moderate depression had a significantly greater rate of remission than patients with severe depression [χ^2^(1) = 7.22, *p* =.007, Figure 2]. Patients with mild-to-moderate depression improved on average by 6.25 U (49.41% of the initial score) on the outcome measures, and patients with severe depression improved on average by 7.90 U (41.06% of the initial score) on the outcome measures. Further, these data suggest an inverse relationship between baseline symptom severity and the likelihood of reaching remission from rTMS treatment. For each unit, decrease in baseline severity scores, the odds of remission (OR) increase by 36% (OR = 1.36). Total number of sessions, length of treatments (weeks), and unilateral or bilateral treatment delivery were not significant predictors of remission.

## Discussion

Initial severity of depression was the only predictor of reaching remission, with patients suffering from mild-to-moderate depression being most likely to remit. Rates of remission may be a consequence of the extent of improvement needed to obtain a PHQ-9 or QIDS-SR score of <5. This suggests a fixed magnitude of improvement on validated rating scales regardless of the ­severity of depression, resulting in differences with overall rates of remission. Though limited by a small sample size, of interest is the determination that the total number of sessions, length of treatments (weeks), and laterality of treatment were not significant predictors of achieving remission.

Numerous studies have reported possible predictors for outcome following rTMS treatment of depression. These predictors include factors, such as anxiety comorbidity, depressive subtype, and duration of the current episode. This study shows a relationship between the severity of the depression and the likelihood of a positive outcome following rTMS treatment. In a limited-scale study, Su et al. (10) reported similar results; however, those researchers studied a patient population that was primarily female (10). Both Su et al. (10) and the current study described above are in contrast to the results reported by Lisanby et al. (3). It should be noted, however, that Lisanby reported only the depression severity for the total sample, without providing a more detailed breakdown of the depression scores and the specific responses to treatment as affected by depression severity. Therefore, it is difficult to explain differences between the three studies.

A growing body of research investigating responses to rTMS treatment suggests underlying physiological characteristics of depression may be the primary predictors of remission. Further research should help clarify which patients may respond best to rTMS and assist with targeted patient selection.

This study had several limitations. This was a limited-scale retrospective study, and the numbers of patients were unequally distributed among mild, moderate, and severe categories. Patients were not distinguished beyond suffering from depression versus major depressive disorder, which may lead to population bias when considering severity. This was a retrospective study analyzing data from a clinical setting, so the application and consistency of treatments were perhaps not as rigorously applied as in a more controlled clinical trial. The data set did not include several clinical factors, such as comorbidity, duration of illness, concomittant medication, types of prior treatments and degree of treatment resistance. Without the ability to consider these other variables, the results showing correlation to severity of depression could be a Type I error.

This study provides evidence that the severity of depression at the onset of treatment with rTMS may be one factor in achieving remission. Further, the total number of sessions, length of treatments (weeks), and laterality of treatment were not significant predictors of remission.

## Conflict of Interest Statement

The views expressed in this article are those of the authors and do not reflect the official policy of the Departments of Army/Navy/Air Force, Department of Defense or U.S. Government. The use of trade names in this publication does not imply endorsement by the authors or the Department of Army/Navy/Air Force, Department of Defense or U.S. Government, nor does it imply criticism of similar products or devices not mentioned. This study did not receive external direct or indirect funding. The authors declare no financial, commercial, or other conflicts of interest.

## Abbreviations

PDMS, patient data management system; PHQ, patient health questionnaire; QIDS-SR, quick inventory of depressive symptomatology; rTMS, repetitive transcranial magnetic stimulation.
